# First assessment of potential distribution and dispersal capacity of the emerging invasive mosquito *Aedes koreicus* in Northeast Italy

**DOI:** 10.1186/s13071-016-1340-9

**Published:** 2016-02-03

**Authors:** Matteo Marcantonio, Markus Metz, Frédéric Baldacchino, Daniele Arnoldi, Fabrizio Montarsi, Gioia Capelli, Sara Carlin, Markus Neteler, Annapaola Rizzoli

**Affiliations:** 1 Department of Biodiversity and Molecular Ecology, Research and Innovation Centre, Fondazione Edmund Mach, Via E. Mach 1, 38010, S. Michele all’Adige, Italy; 2 Istituto Zooprofilattico Sperimentale delle Venezie, Viale dell’Università, 10, 35020 Legnaro Padova, Italy

**Keywords:** *Aedes koreicus*, Climatic factors, Invasive species, Remote sensing, Vector-borne diseases, Ecological modelling, Bayesian inference, Invasive spread

## Abstract

**Background:**

Invasive alien species represent a growing threat for natural systems, economy and human health. Active surveillance and responses that readily suppress newly established colonies are effective actions to mitigate the noxious consequences of biological invasions. However, when an exotic species establishes a viable population in a new area, predicting its potential spread is the most effective way to implement adequate control actions. Emerging invasive species, despite monitoring efforts, are poorly known in terms of behaviour and capacity to adapt to the new invaded range. Therefore, tools that provide information on their spread by maximising the available data, are critical.

**Methods:**

We apply three different approaches to model the potential distribution of an emerging invasive mosquito, *Aedes koreicus*, in Northeast Italy: 1) an automatic statistical approach based on information theory, 2) a statistical approach integrated with prior knowledge, and 3) a GIS physiology-based approach. Each approach possessed benefits and limitations, and the required ecological information increases on a scale from 1 to 3. We validated the model outputs using the only other known invaded area in Europe. Finally, we applied a road network analysis to the suitability surface with the highest prediction power to highlight those areas with the highest likelihood of invasion.

**Results:**

The GIS physiological-based model had the highest prediction power. It showed that localities currently occupied by *Aedes koreicus* represent only a small fraction of the potentially suitable area. Furthermore, the modelled niche included areas as high as 1500 m a.s.l., only partially overlapping with *Aedes albopictus* distribution.

**Conclusions:**

The simulated spread indicated that all of the suitable portion of the study area is at risk of invasion in a relatively short period of time if no control policies are implemented. Stochastic events may further boost the invasion process, whereas competition with *Aedes albopictus* may limit it. According to our analysis, some of the major cities in the study area may have already been invaded. Further monitoring is needed to confirm this finding. The developed models and maps represent valuable tools to inform policies aimed at eradicating or mitigating *Aedes koreicus* invasion in Northeast Italy and Central Europe.

**Electronic supplementary material:**

The online version of this article (doi:10.1186/s13071-016-1340-9) contains supplementary material, which is available to authorized users.

## Background

An increasing number of species is rapidly spreading outside of their original distributional range and invading new territories, gaining the name of invasive species. The factors underpinning invasion processes are numerous and include socio-economical determinants linked to the intensified speed and density of transcontinental commercial and tourist fluxes [1, 2]. Among abiotic factors, anomalous climatic fluctuations [3] and landscape perturbations [4], mainly due to human exploitation of the environment, modify ecological conditions. These altered conditions trigger or facilitate species mixing at various spatial scales, at times resulting in novel ecosystems which are constituted by persistent assemblages of exotic and indigenous species [5, 6]. Among the risks arising from the increased or shifted geographical distribution of species as well as from novel ecosystems [2, 7, 8], the spread rate of infectious diseases is the most pressing for human health [9]. Indeed, many vertebrate and invertebrate species are competent hosts for one or multiple zoonoses –infectious and parasitic diseases transmissible from animals to humans, whose distribution strictly follow the geographical range of their host species [10].

Bloodsucking arthropods, such as mosquitoes, represent the majority of the organisms able to transmit agents of infectious diseases to humans [11]. Indeed they constitute a system able to overpass the skin barrier and deliver the agent of the disease directly into the blood vessels. They use host blood not only as a food source but also to regulate metabolic processes that cause dramatic and key changes in their physiology [12]. Among them, mosquitoes have been the most successful invasive disease-vector group in the 20th century, and are bridge-vectors of infectious pathogens (e.g., arboviruses) which have caused devastating anthropozoonosis. Some arboviruses, such as dengue fever, Rift Valley fever, yellow fever and chikungunya are transmitted by *Aedes* species. These are highly invasive container-breeding mosquitoes, with a native geographical distribution barycentre located in tropical and subtropical regions [13].

During the last thirty years, *Aedes* have spread worldwide, recently becoming pests in several non-tropical countries [14]. In Europe, Italy is the most heavily infested country [14]. Here, the tiger mosquito *Aedes albopictus* (Skuse, 1894) has been recorded for the first time in 1990 [15] and is now well established [16, 17]. This species has been indicated as the primary vector for the first endemic outbreak of Chikungunya in Europe [18]. Furthermore, in France and Croatia *Ae. albopictus* has been blamed for the transmission of the first autochthonous dengue cases reported in Europe (in 2010 and 2013) in the last 80 years [19, 20]. However, in temperate countries, the distribution of *Aedes* species is limited by winter temperature [21, 22], and in Italy, *Ae. albopictus* is mainly present in areas below 600–800 m a.s.l. [23, 24]. In 2011, *Aedes koreicus* (Edwards, 1917), was found in Italy [25]. This species is native of South Korea, Japan, parts of China and ex-USSR countries [26] and was recorded in 2008 in Belgium for the first time outside its native range [27]. *Aedes koreicus* forms a monophyletic *taxon* with *Aedes japonicus* (Theobald, 1901), which is another emerging invasive mosquito in USA and Europe. Given its ecological plasticity, *Ae. koreicus* has been proposed as the next global invasive mosquito species [27, 28], with potential impact on human and animal health as the vector of *Dirofilaria immitis*, a heartworm, endemic in Northern Italy and the Japanese Encephalitis virus, mostly prevalent in Asia [26, 29–31].

According to the few data available about its native distribution, *Ae. koreicus* may be able to tolerate lower winter temperatures than *Ae. albopictus*. It is also better adapted to urban environments than the forest dwelling species *Ae. japonicus* [26, 27]. An exploratory analysis performed using nine data points where *Ae. koreicus* was sampled in its native range (Korea [32, 33]) revealed that its native habitat has a yearly average temperature of 11.5 (sd 0.8) with the minimum average temperature of the coldest month of −9 (sd 1.7; Matteo Marcantonio, personal communication). With the highly competitive species *Ae. albopictus* well established in Italy, *Ae. koreicus* may presumably be outcompeted by *Ae. albopictus* in many areas with mild climate conditions (e.g., through larvae interspecific competition; [34]), but new populations of *Ae. koreicus* might establish in areas too cold for *Ae. albopictus*. Following this scenario, a wider geographical range could be colonized by *Aedes* mosquitoes, potentially widening the spatial distribution of *Aedes*-borne diseases. Therefore, describing *Ae. koreicus* potential distribution is critical for proacting ecological management able to promptly respond to the threat posed by this emerging invasive species [35].

The potential distribution of invasive species in a new geographical area can be assessed through mechanistic or correlative algorithms, generally referred to as invasive Species Distribution Models (iSDMs) [36–39]. The main challenge with correlative iSDM is that, while many spatial modelling techniques require species to be at equilibrium with their environment, emerging invasive species are by definition in a dynamic transition state in the invaded range [9]. The equilibrium assumption may mislead predictions over broad areas since species’ capacities to colonize previously unoccupied areas may affect reliability of model prediction [40]. Therefore, the reliability of some ecological modelling techniques is disputed in the scientific literature, with hybrid (mechanistic together with correlative approach) and adaptive frameworks being more and more explored [39, 41–43]. However, choosing methods is often dictated by more practical reasons such as availability of field and laboratory data, knowledge of species biology, project deadlines and statistical or mathematical expertise. Purely automatic statistical approaches require minimal knowledge about the species’ life history, ecology and physiology, making predictions easy to achieve. By contrast, other approaches make use of such knowledge to select parameters appropriate to model the physiological requirement of the investigated species or build mathematical representations of ecological processes. Such iSDMs require more effort but are more accurate when detailed physiological information are available or in circumstances that require in-depth understanding of survival and spread framework [41, 42].

As part of the LExEM (Laboratory of Excellence for Epidemiology and Modeling, www.lexem.eu) project, we set a network of traps supported by larval searches in northern Italy. Using the collected data, we estimated the potential distribution of *Ae. koreicus* in Northeast Italy with three different iSDM approaches. First, we applied an automatic statistical approach, represented by the Maximum Entropy (MaxEnt) modelling [44]. Second, we implemented a logistic regression model with Bayesian framework informed by using prior knowledge retrieved from literature on the ecologically similar and better studied species *Ae. albopictus*. The information derived from field data is therefore mediated by a-priori ecological information [45, 46]. Third, we applied a Geographic Information System (GIS) physiology-based iSDM, solely relying on known environmental constraints of the species (e.g., the species cannot survive cold winter temperatures, etc.). Beyond describing species distributions, iSDMs have become an important and widely used decision making tool for a variety of applications, such as mapping risk of VBDs as well as their host spread, and determining locations that are potentially susceptible to invasion. Here, making use of multiple iSDMs, we aim to reliably estimate *Ae. koreicus* potential distribution in Northeast Italy, gathering insights into which iSDM should be preferred on the others. Our final goal was to investigate the future expansion of *Ae. koreicus* in the study area combining the developed habitat suitability map with available information about transportation networks and the observed species dispersal rate. Integrating the current and potential distribution of emerging invasive species with their preferred spread pathways is pivotal in identifying the most appropriate strategy to mitigate and control their invasion. In this paper, we hope to provide useful and validated spatial information about *Ae. koreicus* spread to decision makers in order to support control strategies and develop proactive public health policies.

### Study area

**Fig. 1:**
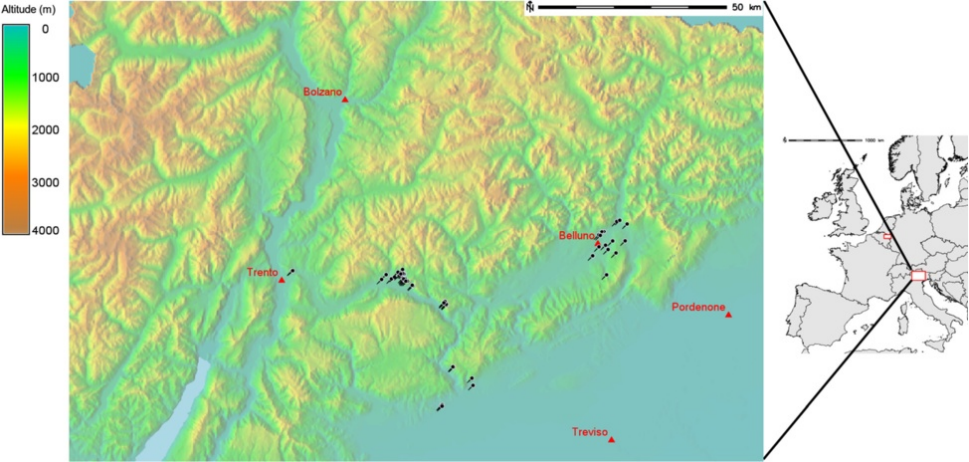
Study areas: Right side: European map with the two red rectangles showing *Ae. koreicus* positive areas for *Ae. koreicus* in Italy (big rectangle) and in Belgium (small rectangle). For the statistical analysis, the Italian study area was used as the training area while the Belgian one as the test area. Left side: Zoom of the Italian study area showing trap locations and major cities, with the shaded digital elevation model as background

The study area is located in Northeast Italy (Fig. 1; latitude N46.75, S45.59, longitude W10.38, E12.82; Datum WGS84). We investigated the presence of *Ae. koreicus* in two administrative units, Trentino and Veneto regions (EU NUTS2 code: ITD2 and ITD3). The area comprises the Eastern section of the Alps and the Northeastern part of the Po Valley. The altitude ranges from 0 to 3,900 m a.s.l. The climate ranges from subartic climate (Köppen climate classification: Dwc) in the Northern-most mountainous part to oceanic climate (Cfc) in the central and southern low altitude and flat part of the study area. The annual average temperature ranges from −6.8 to 15.3, the cumulative annual precipitation ranges from 439 to 1660 mm/year, while the total population count was around 1.7 million with a very variable density, spanning from 0 to 1100 people/km^2^ [47]. The study area includes one of the most developed agricultural, industrial and commercial areas of Italy [25] with dense connections between central and northern European commercial hubs. The area has already been invaded by *Ae. albopictus* which was first detected in 1991 to then gradually colonize all the suitable habitats.

## Methods

### Ethical approval?

#### Data collection

##### Sampling design

We sampled a total of 394 locations from April to November in 2013 and 2014 using various collection methods such as larval searches, ovitraps, CDC-light traps and BG-sentinel traps (Biogents AG, Regenburg, Germany). We derived part of the data from entomological surveillance supported by Veneto Region. The sampling was implemented in order to acquire data on the current distribution of *Ae. koreicus* inside and at the boundary of its known invaded range. The sampled locations were checked every 2 weeks, except for larval search. Eggs collected from ovitraps were maintained in water for hatching, and larvae were reared until the fourth instar for identification [48, 49]. Adults caught in CDC-light traps and BG-sentinel traps were identified and stored at −80 for molecular analysis. Expert entomologists identified each sample at species-level and organized data in a geodatabase. A collection was considered positive (presence) if at least one individual was sampled during the sampling period. The presence/absence dataset was spatially aggregated in a 250 m × 250 m grid aligned to the EuroLST grid [50]. The grid cells containing both positive and negative locations were considered as positive. As a result, a total of 306 grid cells, of which 53 positive and 253 negative, were used in the following statistical analysis.

##### Environmental data sources and model predictors

To model the habitat suitability of *Ae. koreicus*, we used a set of environmental predictors based on a 10-years long average (2003–2012) derived from remote sensing (satellite) data at a spatial resolution of 250 m. Temperature variables have been derived from the EuroLST bioclim dataset, freely available at the EuroLST website (gis.cri.fmach.it/eurolst-bioclim/). Those bioclim variables which integrate temperature together with precipitation were calculated by merging the EuroLST [50] dataset with precipitation data from the Climate Prediction Center (CPC) Morphing algorithm (CMORPH) Version 1.0 [51] which we calibrated against data from the Global Precipitation Climatology Project (GPCP) Version 2.2 [52]. Moreover, two additional temperature predictors were derived; average temperature of the coldest month (*TavgCM*) and average temperature of mosquito growing season (*TavgGS*). The two latter variables were considered in order to match the biology of *Aedes* spp. with temperature variables. Indeed, in the study area, the time period from April to September represents the most favourable conditions for *Aedes* population growth. Furthermore, cold temperatures under 0 are a limiting factor for diapausing egg survival. On the one hand, in the literature it is reported that *Aedes* cold-acclimated and diapausing eggs can survive very low temperature (−10/-12) for a brief to moderate period of time [53, 54]. On the other hand, long periods of average cold weather represent a chronic stress which may more strongly limit the fitness (e.g., hatching) of diapausing eggs. Therefore, the average temperature of the coldest month may be more effective to limit mosquitoes potential distribution than the average minimum temperature of the coldest month.

We obtained data on the vegetation biomass from the MODIS Normalized Difference Vegetation Index (NDVI; MOD13Q1) product [55]. Vegetation indices, as NDVI, have been extensively used to describe disease risk and habitat suitability for different species of mosquitoes [56–58].

Given that the ecosystem water content might also limit mosquitoes habitat suitability, as water is a key component of their ecological niche, we calculated the Normalized Difference Water Index (NDWI; [59]), derived from the MODIS surface reflectance product (MOD09A1). NDWI and NDVI embed different wavelengths, and so they should be considered as complementary [59]. We averaged NDVI and NDWI values pixel-wise in four seasonal groups over a three month period each (January-March, April-June, July-September, October-December), in order to have a representation of the vegetation coverage and ecosystem water for each of the four seasons.

The initial set of 29 environmental predictors (19 EuroLST/CMORPH bioclim, 2 further temperature-based variables, 4 seasonal NDVI and 4 seasonal NDWI; Table 1) was used in different combinations as input for the modelling approaches described in detail in the next section. All the considered environmental parameters have already been shown as relevant for mosquito iSDMs [60].

**Table 1:** Description of the predictor variables. We reported source and spatial resolution of each group of predictor variables

N	Variable	Source	Spatial resolution
19	Bioclim 1–19*	MODIS LST/CMORPH	250 m
1	Avg. T growing season	MODIS LST	250 m
1	Avg. T coldest month	MODIS LST	250 m
4	seasonal NDWI	MODIS LST	250 m
4	seasonal NDVI	MODIS LST	250 m

* http://www.worldclim.org/bioclim

### Modelling framework

SDM are a set of algorithms which quantitatively describe areas that support the presence of a given species, based on experimental data, known presence/absence data and the associated environmental conditions. These models seek, despite some limitations, the species ecological niche in the Hutchinsonian sense [61]. We made use of three different modelling approaches to estimate *Ae. koreicus* potential distribution in Northeast Italy. These three techniques rely on automatic statistical methods or on physiological knowledge of the species life history cycle. The target output of all these three modelling techniques was an environmental suitability indicator, expressed as a continuous value from 0 (no suitability) to 1 (complete suitability). We associated each suitability value to its respective EuroLST grid cell, at a resolution of 250 m. Therefore, we visualized the predicted environmental suitability (habitat suitability) in potential distribution maps.

### Maximum Entropy (MaxEnt) approach to species distribution modelling

MaxEnt is a common modelling framework used in species distribution modelling [44, 62]. MaxEnt minimizes the relative entropy between the probability density of the predictors estimated from the presence data and the probability density of the predictors estimated from the region of interest (background information). This means that the geographic extent and the number of background samples influence the results. For each predictor, response curves can be generated describing how predictor values are related to the estimated suitability.

After having tested different buffer sizes, we have placed a buffer of 5 km around the presence data, representative of locations accessible for *Ae. koreicus* via dispersal and which approximate the overall study area environmental conditions [63, 64]. The resultant region has been used as input for MaxEnt modelling. We used MaxEnt as machine learning algorithm, letting it decide which predictors were important through regularization [65]. Therefore, we ran the MaxEnt model without any previous biologically-based selection of the predictor variables. However, since several predictors were highly correlated among them, we performed a correlation analysis in order to limit multicollinearity issues. We excluded all those predictors showing a correlation higher than 0.50 (Pearson’s *r*). The predictors exclusion was performed selecting the most correlated couple, followed by a random draw to decide what to exclude of the two predictors. We carried out all the analysis in R [66], using dismo [67] package.

### Bayesian logistic regression (logBAY) with Markov Chain Monte Carlo simulation

Logit-link Generalized Linear Models (GLMs) are standard regression methods to model habitat suitability in ecology [68]. The presence or absence of a species is transformed in a probability function, real number in the range [0, 1], through a logistic transformation of the presence/absence odds (*log*(1/1  −  *p*)). We wrapped the logistic regression in a Bayesian framework using Just Another Gibbs Sampler (JAGS) [69] in combination with rjags [70] and coda [71] R packages. We used presence or absence data as response variable, while as predictor variables we chose those environmental variables with the strongest credibility in shaping *Aedes* ecological niche, as follows: *i)* average temperature of mosquito growing season *TavgGS*, *ii)* average annual temperature (*TavgY*), *iii)* average of the minimum temperature (*TavgM*); *iv)TavgCM*; *v)* cumulative annual precipitation (*PcumY*) and *vi)* spring NDWI (*NDWIavgS*) [14, 21, 22, 27, 28, 49, 54, 72–77]. All predictors were scaled using their mean and standard deviation as follows: (*x*  −  *mean*(*x*))/*sd*(*x*), where *x* is the predictor variable. Even though the role of precipitation as limiting factor for container-breeding mosquitoes is controversial [73], we included it among predictors since water availability affects the aquatic stages of the mosquito life cycle [74]. Moreover a preliminary exploratory analysis showed a high correlation between precipitation and *Ae. koreicus* presence/absence for our dataset. Nevertheless, the observed correlation may be a spurious pattern linked with a different detectability probability in different parts of the precipitation range [78].

We used Gaussian distributed informed priors derived from [22] for the temperature based variables, whereas non-informative priors (normal distribution with mean = 0; precision = 10*E*  −  12) for all the other variables (Table 2). To select the combination of variables carrying the most information on mosquito distribution, we run all the models possible combining the six aforementioned predictor variables (including models with interactions between *TavgGS* or *TavgY* and *PcumY* and models with *TavgY* second order polynomial function). Each model was initialized using maximum likelihood estimates for each coefficient and 10000 burn-in Markov Chain Monte Carlo (MCMC) iterations to find a good starting point to sample a representative Posterior Probability Distributions (PPD). The models were therefore ranked using the Deviance Information Criterion (DIC) with 1,000 MCMC iterations and thinning set of 5. The most informative model (lowest DIC) was used to sample 10,000 times with thinning set of 50 the PPD of model parameters and of *Ae. koreicus* occurrence in each pixel of the study area. The convergence of MCMC chains was monitored using Gelman and Rubin’s convergence diagnostic between two MCMCs [79]. The PPD Highest Density Interval (HDI) was calculated using the function proposed in [80]. The average value of PPD was assigned to the correspondent pixel, resulting in the habitat suitability map for *Ae. koreicus*. Furthermore, the uncertainty linked to the average pixel prediction was assessed (and mapped; not reported) deriving the 95 % Bayesian confidence interval of the PPD of each pixel. We reported all the steps to reproduce the logBAY model as an R function in the Additional file 1.

**Table 2:** Average and precision for informed and non informed priors. The precision of a distribution is the inverse of its standard deviation

Predictor	Average	Precision
*TavgGS* ^a^	2.580	0.835
*TavgCM* ^a^	1.9623	0.654
Others	0	10e–12

^a^Values from [22]

### GIS physiology-based (PHY) suitability modelling

This iSDM approach considers environmental parameters corresponding to physiological constraints of *Ae. koreicus*. The exact physiological constraints for this species are currently unknown. We used a conservative and parsimonious approach by assuming that the same environmental parameters which represent limiting factors for *Ae. albopictus* can be applied, most importantly: the average temperature of the coldest month (*TavgCM*) and the average temperature of the hottest quarter of the year (*TavgHQY*). The temperature of the coldest month determines overwintering suitability: if the coldest month is under a certain threshold, diapausing eggs will not survive and a persistent population can not be established. If the temperature of the hottest quarter of the year does not reach a certain value, larvae can not develop and adults can not reproduce. Additionally, precipitation can determine habitat suitability, but needs to be treated with caution because irrigation and small anthropogenic water reservoirs can compensate for low precipitation. Other environmental parameters of potential importance are the average temperature of the mosquito growing season (*TavgGS*) and annual average temperature (*TavgY*). *TavgY* has previously been used to model habitat suitability for *Ae. albopictus*, but can not be linked to a particular physiological constraint. Suitable summer temperatures might be averaged out by cold winter temperatures, and equally cold winter temperatures might be averaged out by hot summer temperatures. The specific threshold for the three environmental parameters (average temperature of the coldest month, average temperature of the hottest quarter of the year and annual precipitation) was estimated from the values observed at sampling locations with presence of *Ae. koreicus*. We used the lower bound of the 99 % confidence interval as the low threshold for environmental parameters (Table 3). The estimated thresholds were used for habitat suitability modelling resulting in suitability maps. All temperature thresholds were transformed with a sigmoid function such that zero means not suitable, 0.5 corresponds to the actual threshold and 1 means highly suitable (Appendix C). A margin of 4 was applied to the sigmoid function for temperature. Moreover, for annual precipitation (*PcumY*), a margin of 200 mm/year was applied. Compared to MaxEnt, we defined a priori response curve for relevant environmental parameters, whereas such response curves are a diagnostic result of MaxEnt. The separate suitability indicators were multiplied in order to obtain a single general suitability index where 0 means that any single parameter was 0 (not suitable) and 1 represents that all single parameters were 1 (highly suitable).

**Table 3:** Predictor variables used in the GIS physiology-based suitability model. The descriptive statistics refers to the location of all the positive traps in the study area

Parameter	Average	Standard deviation	Lower bound - 99 % CI
*TavgHQY* (°C)	21.41	10.76	18.63
*TavgCM* (°C)	0.38	1.33	−3.06
*PcumY* (mm/year)	1182	105	912

### Model performance accuracy

Model performance accuracy was measured assessing the error rate as a percentage (i.e. error rate (%) equals the number of incorrect cases divided by the total number of cases tested), as well as Cohen’s kappa coefficient (k), which is a measure of agreement that takes into account chance effects [81] and True Skill Statistics (TSS; [82]), an accuracy index not sensitive to prevalence. The optimal thresholds to discriminate the continuous model outputs in the presence or absence category were estimated by maximising sensitivity together with specificity.

We performed a further qualitative validation of the models sensitivity predicting *Ae. koreicus* overall average suitability and standard deviation in the only other known invaded area in Europe, the Maasmechelen municipality in Eastern Belgium (Fig. 1). In this locality, a viable hibernating *Ae. koreicus* population persists in a homogeneous 6 km^2^ industrial area since its first detection in 2008 [27]. When dealing with emerging invasive arthropods, absence points have a high likelihood to represent areas where the trap failed to catch entities of the species despite presence in the area, or areas that are inside the species ecological niche, but which have not yet been invaded (i.e., dispersal limitation). Therefore, we emphasized model sensitivity on specificity since it should be considered more effective to assess iSDM predictive power [83–85].

### *Aedes koreicus* spread analysis

To estimate the *Ae. koreicus* spread rate since introduction in the study area, we used information about its presence since 2011, when it was recorded for the first time in a small village near Belluno (Sospirolo village, latitude N46.14; longitude E12.07, datum: WGS84) [25]. In absence of information about the presence of the species before 2011, we assumed this geographical location as the centre of gravity for the introduction point(s).

In addition, we calculated the centre of gravity for the coordinates of positive traps for 2013 and 2014 respectively. We assumed that the range expansion has been constant through time, therefore we divided the Euclidean distance between 2011, 2013, 2014 centres of gravity by 4, representing the years since the introduction, deriving an approximate spreading rate, defined as spread distance in kilometres per year. Afterwards, we built a road network, weighted by the travelling distance between each road segment and the introduction point (root of the network, assumed to be the village Sospirolo). The road network was acquired from the OpenStreetMap project (openstreetmap.org), cleaned from tertiary roads, tracks and pathways which were assumed to be of low importance for mosquito dispersal. All the unconnected (not connected to the root of the network) road segments were also removed from the network.

The following step was to intersect the weighted road network with the habitat suitability map, to derive a distance-suitability weighted cost network. For the reference suitability map, we chose the one derived by the iSDM with the best predictive performance. This step was carried out to increase the cost for those locations that, despite being spatially close to the introduction location, were ecologically distant from *Ae. koreicus* ecological niche. We assumed that, for high suitability values (defined using the suitability threshold at which sensitivity plus specificity were maximized), the cost for the spread of the mosquito was the distance from the introduction point divided by the suitability value, while for suitability values below the threshold, the new weighted distance from the introduction location was the original distance from the introduction location divided by the suitability values raised to the power of 1.5 (penalty derived from empirical observations of the invasion process).

Eventually, we split the distance-suitability weighted road network into invasion cost isolines according to the observed *Ae. koreicus* dispersal rate. This step was performed in order to estimate areas with the same probability to be invaded in a defined temporal span (in years).

All the spatial analysis were performed using GRASS GIS 7 [86] modules (particularly *v.net* tool set).

## Results

**Fig. 2:**
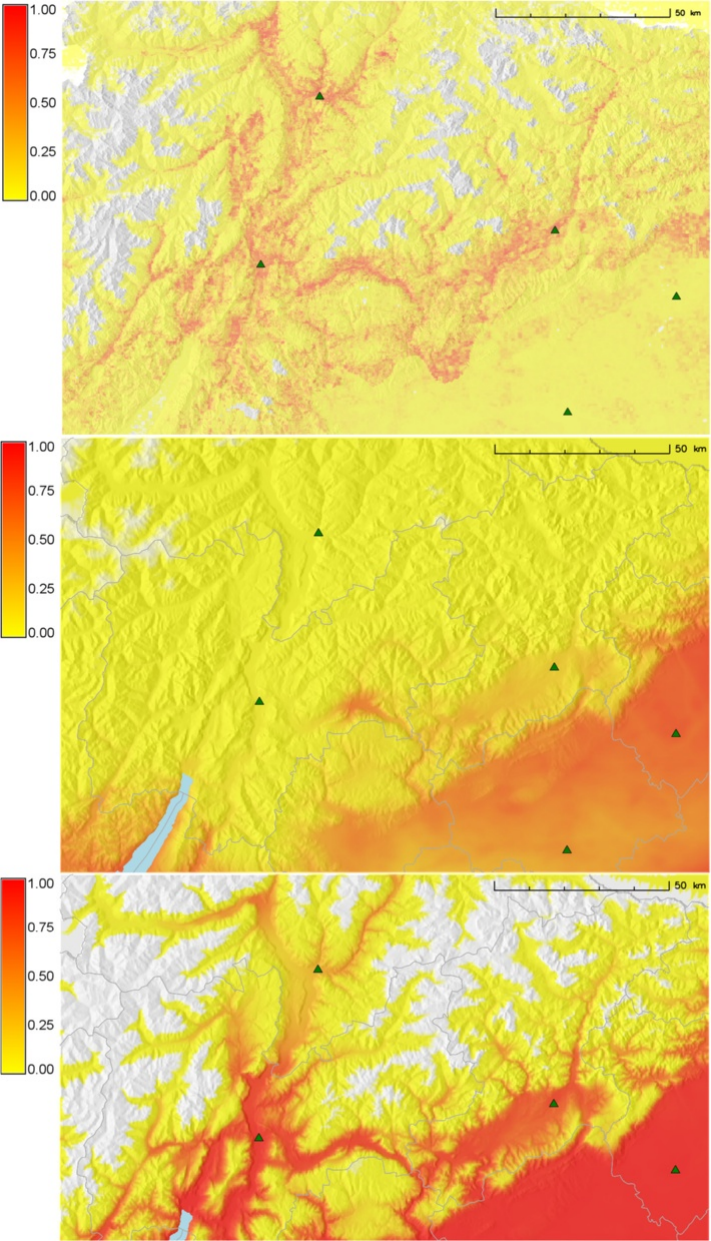
*Ae. koreicus* potential distribution maps: The values range from 0: no suitability; to 1: complete suitability. The green triangles represent the centroids of the main cities in the area

The potential distribution maps for *Ae. koreicus* derived from the three models are reported in Fig. 2.

### MaxEnt modelling results

The output of the correlation analysis, which was a matrix with 9 predictors (Appendix A), was used as an input in the MaxEnt model.

Variable importance can be estimated by different means: percent contribution, permutation importance, and jacknife gain. These three measurements provide different rankings and are reported in Appendix A. According to the rank sum of the three different criteria, the two most important variables for the MaxEnt model were temperature seasonality (bio4) and maximum temperature of the warmest month (bio05). The response curve of bio4 suggests that *Ae. koreicus* presence probability is relatively constant until it drops suddenly in the localities where seasonality becomes extreme. Bio5 response curve implies a monotonic increase of presence probability in a temperature range between 22 and 28 °C, after which it assumes an asymptotic trend (Appendix A).

The *Ae. koreicus* potential distribution map derived from the MaxEnt model showed values ranging from 0 to 0.94, with an average suitability of 0.11 (Fig. 2a). According to the MaxEnt model, suitable areas are concentrated along the main Alpine valleys.

### Bayesian logistic regression modelling results

The best logBAY model (lowest DIC; Table 4) comprised the average temperature of the growing season (April to September; *TavgGS*), the minimum temperature of the coldest month (*TavgDEC*) and the cumulative annual precipitation (*PcumY*). All the predictors were positively correlated with the presence of *Ae. koreicus*. The PPD of the model coefficients with their mean and 95 % HDI is showed for the best model in Appendix B. The 95 % HDI of *TavgGS* and *PcumY* did not include 0, meaning that the credible values of these model parameters are different than 0 (Appendix B).

**Table 4:** Model specifications and DIC for the best 15 logBAY models plus the full model

N	Model terms	DIC
1	*TavgY*	272.0
2	*PcumY* + *NDWIs*	264.0
3	*TavgDEC* + *PcumY*	222.0
4	*TavgDEC* + *PcumY*	222.0
5	*TavgM* + *PcumY*	220.2
6	*TavgY* + *Tmin* + *PcumY*	220.0
7	*TavgGS* + *TavgY* + *TavgM* + *TavgDEC* + *PcumY* + *NDWIs* (full)	218.2
8	*Tmin* + *PcumY*	217.0
9	*TavgY* + *PcumY*	215.4
10	*TavgY* + *PcumY*	215.0
11	*TavgY* + *PcumY* + *NDWIs*	215.0
12	*TavgY* + *Tmin* + *PcumY* + *NDWIs*	212.0
13	*TavgGS* + *Tmin* + *PcumY* + *NDWIs*	211.6
14	*TavgGS* + *Tmin* + *PcumY*	210.8
15	*TavgGS* + *TavgM* + *PcumY*	209.5
16	*TavgGS* + *TavgDEC* + *PcumY*	208.3

The logBAY suitability map, built using the average of the PPD, showed values ranging from 0 to 0.83, with an average suitability of 0.14 (Fig. 2b). The suitability predicted by logBAY model cut the study area in two distinct sections: high suitability in the southern part, low suitability in the northern mountainous area. The highest suitability was indicated for Pordenone, Treviso provinces and on the surroundings of lake Garda.

### Physiology-based modelling results

The PHY suitability surface resembles the one of MaxEnt, with the difference of a much higher absolute suitability value. By definition, the PHY suitability value for most known presence sites is one. The main difference between the PHY, logBAY models and MaxEnt is due to the upper threshold in the MaxEnt response curves, which are mainly composed of parameters derived from temperature, whereas the other two models have not imposed an upper threshold on temperature (logBAY is a linear regression model, while for PHY there is no physiological restriction for maximum monthly temperature in the study area, which rarely reaches more than 35 °C).

The suitability values predicted by PHY ranged from 0 to 1, with an average suitability of 0.32. PHY had the maximum average suitability value among the developed models, with more than 65 % of the studied areas having values higher than 0.50 (Fig. 2c). Very high suitability was assigned to Po, Adige, Valsugana and Sarca valleys. Furthermore Piave, Isarco as well as other minor valleys were characterized by moderate to high suitability values.

### Model validation

The suitability threshold at which sensitivity plus specificity were maximized for each model is reported in Table 5 together with Kappa statistics, TSS and percent error rate. The threshold at which sensitivity plus specificity were maximized varies considerably between the three models. After grouping the suitability values in suitable and not suitable classes using these thresholds, MaxEnt reported the highest error rate. On the contrary, logBAY and PHY had TSS and Kappa values indicating from substantial to almost perfect agreement with observed data [87]. PHY showed the highest sensitivity while logBAY the highest specificity.

**Table 5:** Model performance accuracy. We reported the suitability thresholds at which sensitivity plus specificity were maximized, Kappa statistics, TSS and error rate for each of the model

Model	Optimal Threshold	Kappa	TSS	Predicted high - Presence	Predicted low - Absence	Error rate (%)
MaxEnt	0.62	0.55	0.13	33/53	128/253	47.4
logBAY	0.14	0.84	0.69	50/53	189/253	21.9
PHY	0.71	0.70	0.45	50/53	129/253	41.5

Applying the discriminant thresholds listed in Table 5 to the suitability maps, we found that 3 %, 26 % and 30 % of the study area was reported as suitable by MaxEnt, logBay and PHY models respectively.

**Fig. 3:**
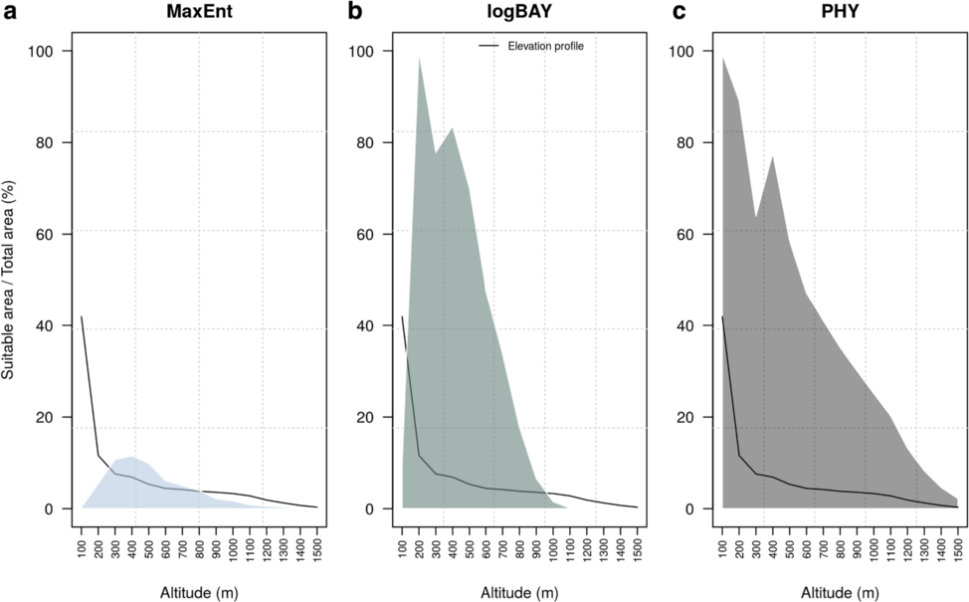
Altitude profile of suitable area: This figure depicts the percentage of suitable area over the total area for each altitude class for **a**) PHY; **b**) logBAY and **c**) MaxEnt model. The black line represents the percentage of area in each corresponding altitude class

The result of the cross-tabulation between elevation and suitable area is reported in Fig. 3. All the models agreed on the suitable area being concentrated at low altitude (0–800 m). Half of the total area between 0 and 800 m was indicated as suitable according to logBAY and PHY. It is interesting to note that all the profiles in Fig. 3 show a spike in suitability around 400–500 m. Moreover, PHY model predicted as suitable a remarkable percentage (16 %) in higher altitude area (above 800 m).

To further validate the model using an independent set of data, we applied each model to the only other area invaded by *Ae. koreicus* in Europe: Maasmechelen municipality in Belgium (Fig. 1). We reported the descriptive statistics of the predicted suitability distribution in Table 6. PHY model predicted high suitability, whereas logBAY very low suitability.

**Table 6:** Descriptive statistics for the distribution of suitability values in Maasmechelen municipality, Belgium, for all three models

Model	Avg suitability	Min suitability	Max suitability
MaxEnt	0.46	0.03	0.70
logBAY	0.10	0.07	0.14
PHY	0.61	0.38	0.78

### *Aedes koreicus* spread analysis

**Fig. 4:**
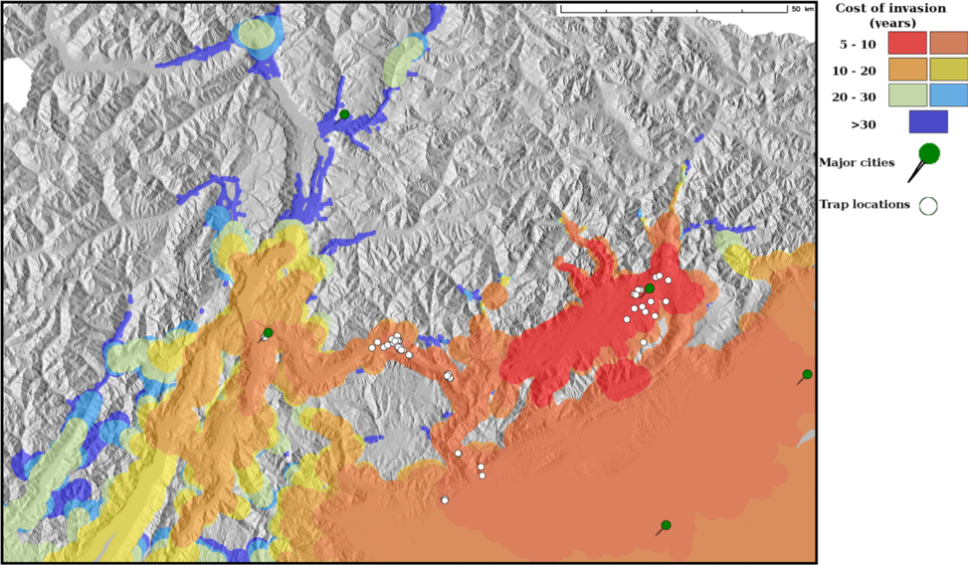
Potential spread of *Aedes koreicus* predicted through road network analysis: Areas with the same cost of invasion are displayed using a red-green-blue colour scale. The cost of invasion is expressed in years since the species’ introduction (2011). Cost of invasion is a function of the travelling distance from the introduction point based on the observed rate of shift of the invaded range centroid and the predicted habitat suitability. Major cities (green pushpins) and sampling locations (white circles) are also reported

From 2011 to 2014, the average shift of the invaded area centroid was approximately 8 km/year. The analysis performed to predict the spread of *Ae. koreicus* showed that the most likely dispersal direction was along Valsugana Valley (Fig. 4). Furthermore, the mosquito may be already present in the northern part of Verona province (south-east of the study area; predicted to be invaded within 5–10 years after introduction). According to our results the mosquito spread might be rather fast in the southern part of the study area due to both the dense road connection with the introduction point and to the high habitat suitability. Even though the dispersal along the highly populated Adige Valley was found to be slower than in the southern part of the study area, probably due to its more rough topography, overall it will potentially be at high risk of invasion during the forthcoming decades.

## Discussion

*Aedes koreicus* is an emerging invasive species in Europe, and is a nuisance and potential vector of infectious diseases [26, 31]. In this study, we assessed its habitat suitability in Northeast Italy, making use of iSDMs and a limited amount of field data supported by prior knowledge from related species. We also investigated the potential pathways and timing of future spread through a road network analysis. The main outcome was that the known distribution of *Ae. koreicus* is only a fraction of the potentially suitable area. However, we observed a rather variable characterization of the suitable area, highly dependent on the considered iSDM. MaxEnt predicted the smallest suitable area, while PHY the largest. The observed differences are mainly due to the lack of upper temperature threshold limits for logBAY and PHY (Appendix A, C), which allows high suitability in the warmest section of the study area. Despite potential limitations due to the approximation of biological patterns with linear relations in ecological niche modelling, the lack of upper thresholds for temperature should not to be considered an artefact due to the studied species and the climatic characteristic of the study area. Indeed, *Ae. koreicus*is a temperate-continental *taxon* and the studied area is characterized by monthly average temperatures rarely exceeding 30 °C (therefore well inside the temperature niche of other *Aedes* species; [88]). However, high temperatures might affect local abundance of *Ae. koreicus* (e.g., [89] observed a decrease of *Ae. japonicus* larvae survivorship at temperatures over 22 °C under laboratory conditions).

All the models were mainly driven by temperature variables, confirming what has already been found in literature for other *Aedes* species (e.g., [90]). The most important predictors for MaxEnt were bio4 and bio5, the best logBAY model included two temperature variables *TavgGS*, *TavgDEC* in addition to *PcumY*, while PHY was completely constrained by temperature (the included precipitation threshold was always exceeded in the study area). Mosquitoes are small-bodied poikilotherms, meaning that ambient temperature is the main abiotic factor limiting their ecological niche and, therefore, their geographical distribution [77, 91]. High temperature decreases embryonic (e.g., [92]) and larval (e.g., [93]) development time, and the size of adults (e.g., [94]), while cold winter temperatures have a severe impact on the survival of diapausing eggs [54]. The lesser importance of environmental indicators other than temperature can be explained by the considered spatial scale (i.e., extension and grain), where vegetation variability might be less important than climatic conditions [95], and by the autoecology of *Aedes* mosquitoes, container-breeding species, able to develop independently of the regional precipitation trend and environmental variability. At a finer spatial scale and in urban habitats, vegetation may be more influential on *Aedes* life cycle. Indeed, in this setting, even small pockets of vegetation favour habitat heterogeneity, allowing mosquitoes to regulate extreme weather conditions [96].

We performed model validation with a two-step analysis, i.e. a classical accuracy assessment with a dependent set of data and a qualitative sensitivity analysis using an independent set of data. The classical accuracy analysis suggested logBAY as the best model. However, PHY indicated a moderate-to-high agreement with observed data, while MaxEnt performed poorly. All the three models predicted presence locations with high accuracy (high sensitivity). On the contrary, model specificity was relatively low for all three models. As extensively reported in the literature, absence data is the Achille’s heel of SDM due to the high uncertainty linked to absence data veracity (see [97–99]). This is especially true when dealing with emerging invasive arthropods, whose true absence is hard to identify (see [84, 85]). Therefore, to further assess the model accuracy, we decided to perform a further sensitivity analysis. MaxEnt and logBAY predicted low suitability for Maasmechelen municipality, despite this, the area has hosted a viable *Ae. koreicus* population at least since 2008 [27]. By contrast, PHY model predicted a moderate to high average suitability. This model, based on the construction of mechanistic overlay functions for climatic constraints, is partially independent from local datasets and thus tends to be more accurate for prediction on an independent dataset.

The percentage of the study area predicted as suitable varied from 3 to 30 %, encompassing different topographic and environmental conditions. To better characterize the predicted suitable area, we cross-tabulated it with a digital elevation model. PHY and logBAY indicated most of the low-altitude areas as highly suitable, while MaxEnt showed a peak in suitability distributed around moderate altitude (400 m). This outcome may be due to the buffer size (5 km) chosen around the presence points to derive background data, which may over-represent hilly areas, influencing the MaxEnt output. However, all three models showed a peak in suitability around 400–500 m, which may indicate optimal ecological conditions for *Ae. koreicus*. In support of this hypothesis we noted that the trap with the highest *Ae. koreicus* abundance was located at an altitude of 451 m. Another interesting outcome is that PHY predicted as suitable areas between 600 and 1500 m. This altitudinal range represents a still empty niche for invasive *Aedes*, as 600 m is the altitudinal limit for *Ae. albopictus* distribution [24] in Northeast Italy. As a result, the area between 600 and 1500 m suitable for *Ae. koreicus* should be particularly monitored as, here, the invasion would not be constrained by biotic interactions with species with similar evolutionary traits.

The accuracy assessment indicates PHY as the model with the highest prediction power, being in moderate-to-high agreement with observed data in the study area and predicting high suitability in a positive location with a different environmental setting. Therefore, PHY model was chosen as reference to investigate how *Ae. koreicus* may further spread in Northeast Italy. The spread analysis was achieved considering the observed dispersal rate since introduction, the preferred dispersal pathways, study area connectivity and habitat suitability. *Aedes* species have a short flight range, with a flying dispersal capability of 200–300 m radius per week around the hatching location [100, 101]. However, the short active dispersal range is generally compensated for by long distance used-tyres transportation and the plant nursery trade (*Dracaena* sp.) in the form of drought resistant eggs [102]. The local dispersal in new invaded areas is also boosted by humans, through the movement of garden waste, moist vegetation and water containers that can hold eggs or larvae as well as dispersal in trucks transporting used tyres or private vehicle [102, 103]. As a consequence, it can be inferred that the local dispersal probability in a newly invaded area is a function of the introduction point, local transportation network as well as habitat suitability. From these premises, we derived a distance-suitability weighted road network to predict which areas in Northeast Italy have the highest probability to be invaded. The results revealed how the centroid of the invaded range has been shifting approximately 8 km/year since 2011 (putative introduction year). Assuming a constant invaded range shift and driving it along the shortest road pathway (lowest cost from the introduction point), weighted according to the suitability of each road segment, we built a potential dispersal network which represents a reliable dynamic description of the invaded area evolution in the next decades. The simulated spread predicted all the known presence locations (except one) as invaded in a time frame of 5–10 years since its introduction. Moreover, it showed how the species may have already invaded the two major cities in the southern part of the study area, Treviso and Pordenone. However, a first investigation in July 2015 did not find positive locations in these cities [104]. Furthermore, the simulated spread predicted the north part of Po Valley and the southern Adige Valley as invaded in the next decade. A favourable topography (continuous flat areas), mild climate and dense and congested road network underlie the predicted rapid spread in these parts of the study area. On the contrary, we noticed no predicted spread in the north side of the study area, apart from limited spots such as the southern Isarco and northern Adige Valleys, where temperature hotspots due to towns (Urban Heat Island) as well as high road connectivity may favour *Ae. koreicus* spread over the next years.

The simulated spread is a reliable approximation of the future expansion of *Ae. koreicus* distribution range since it integrates a validated suitability surface as well as the most likely dispersal pathways at local scale. A partial validation of the adopted spread analysis comes from a similar study on *Ae. albopictus* by [93]. The authors found that *Ae. albopictus* is currently surfing a dispersal wave in Southern France, with occasional “jumps” that did not result in new colonization fronts. However, it should be remembered that the proposed approach may underestimate the dispersal rate. This is due to the choice to consider the centroids as indication of the invaded area shift as well as the deterministic nature of our approach which does not integrate stochastic events such as occasional introductions in spatially distant but ecologically close locations. Stochasticity in species distribution change underlies unpredictable events that sometimes strongly boost species dispersal and colonization of new areas. Besides, in the southern part of the study area, biotic interactions and out-competition by *Ae. albopictus*, not considered in this study, may slow down *Ae. koreicus* spread [34], modifying the outcome of the invasion process. Preliminary larval competition experiments suggested that the larval development of *Ae. koreicus* might be negatively affected by the presence of *Ae. albopictus* (Frédéric Baldacchino, personal communication).

## Conclusion

 Despite the rising concern about biological invasions after recent economic and human health issues due to invasive species (e.g., *Drosophila suzukii* (Matsumura, 1931) and *Xylella fastidiosa* (Wells et al. 1986) as crop pests and *Ae. albopictus* as a vector of tropical pathogens; [18, 105, 106]), at present there is no coordinated plan which aims to manage *Ae. koreicus* in the study area. Multiple control and mitigation strategies are available to eradicate, mitigate or control invasive species [107]. At the beginning of an invasion, as is the case of *Ae. koreicus*, the most effective control strategy is through inspections followed by destruction of removable breeding sites (e.g., plastic drums) and treatment with larvicidae of fixed sites (e.g., concrete bins). This strategy is time consuming and might be improved in terms of cost-effectiveness by targeting the most productive breeding sites. However, there is often a limited understanding of the biology of emerging invasive species and, consequently, of the hazard they represent [108]. Delays in early mitigation actions result in escalating costs of control, reduced economic returns from management actions and decreased feasibility of management [35, 109, 110]. iSDMs and spread pathway analysis are powerful tools to shed light on the present and future invader distribution and to inform on-ground control of the invasions [111]. We suggest that modelling and mapping the spatial distribution of invasive mosquitoes, validated by entomological surveys, should routinely support the implementation of control actions to limit their expansion. We hope that the results in this study serve as a foundation for design policies aiming to limit *Ae. koreicus* invasion in Northeast Italy.

## Figures and Tables

**Fig. 5:**
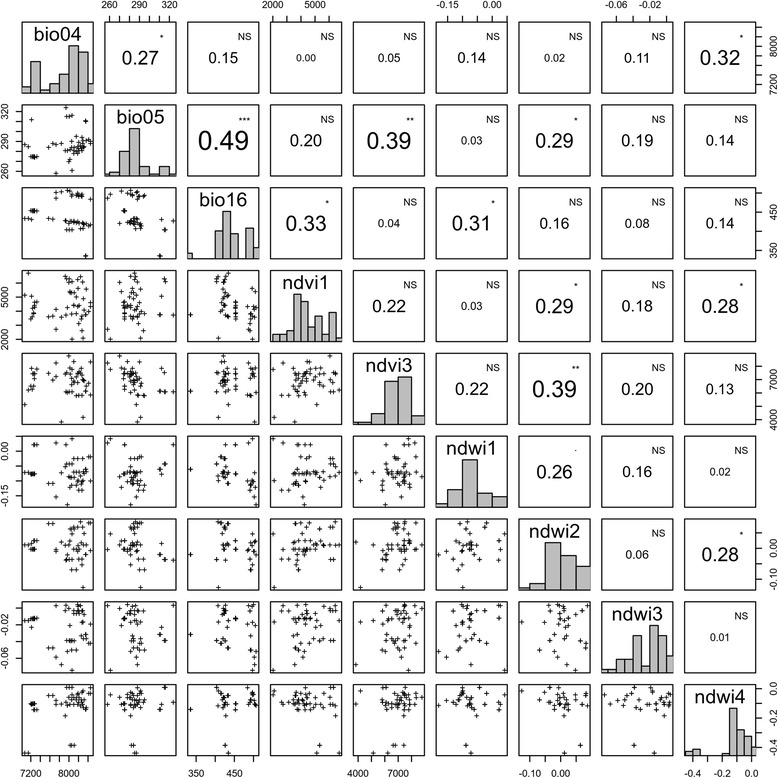
Correlation Matrix: The correlation matrix for the predictors set, input of MaxEnt model

**Fig. 6:**
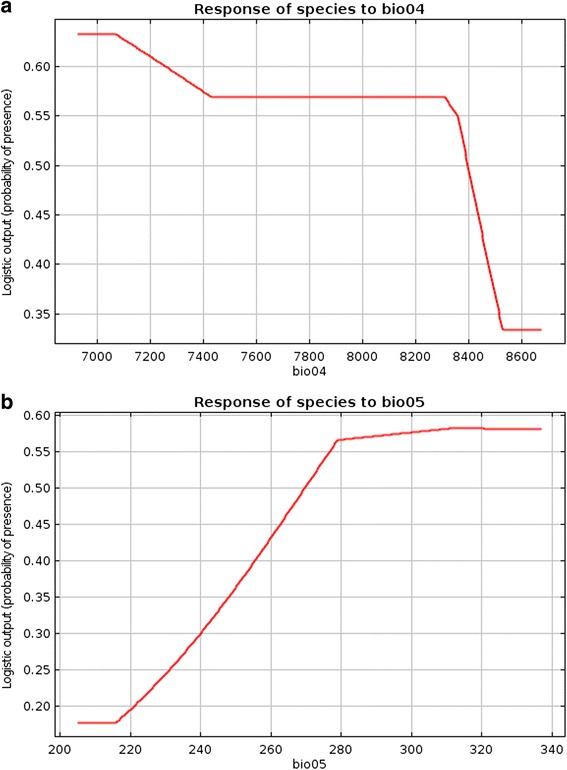
MaxEnt Response Curves: MaxEnt response curve for the two predictors with the highest overall ranking (as calculated in Table 7). **a**) MaxEnt response curve for bio04. **b**) MaxEnt response curve for bio05

**Fig. 7:**
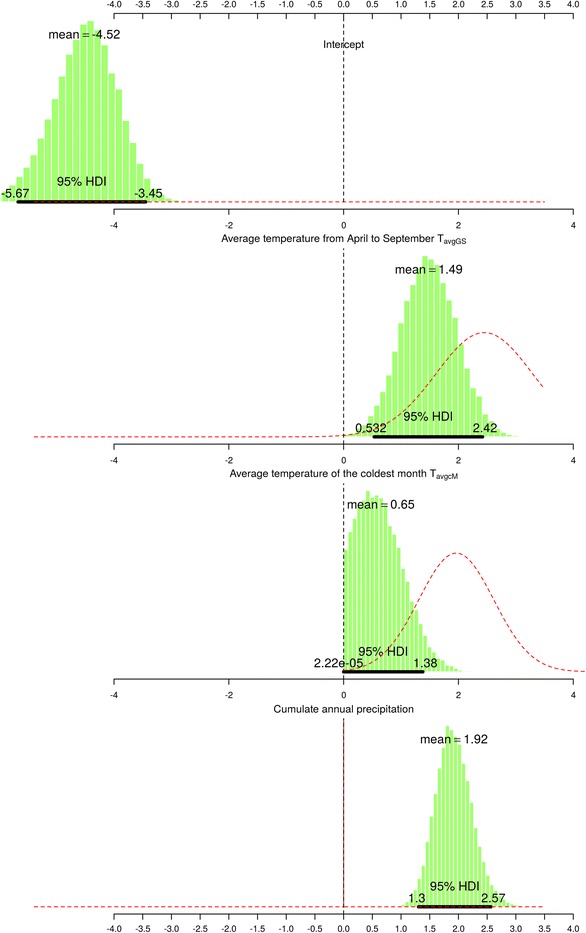
Posterior Probability Distribution for the best model parameters: The red dashed lines represent the distribution of the priors while the black horizontal line is the 95 % High Density Interval of the distribution. The distribution average, lower and upper bound of 95 % HDI were also reported in the figure

**Fig. 8:**
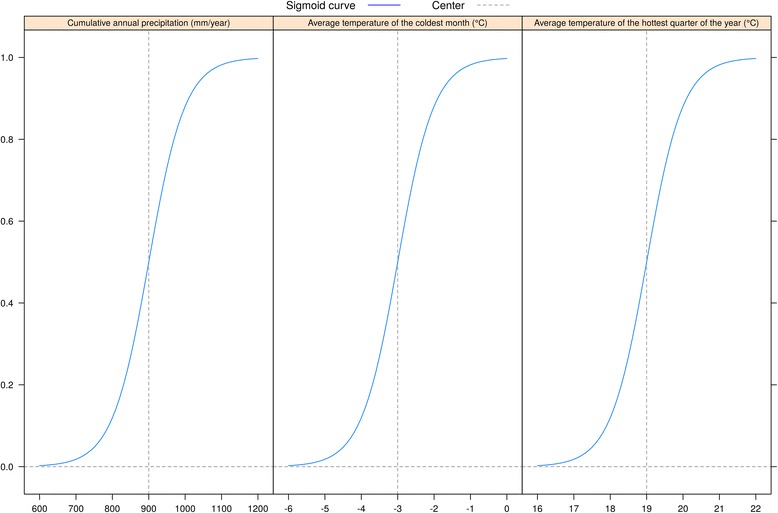
Sigmoid curves for PHY model: We present the three sigmoid functions used to transform the environmental parameters to separate suitability indices

## Appendix A: Correlation matrix and MaxEnt response curves

**Table 7:** Ranking of the 5 most important variables for MaxEnt model. We assigned a score ranging from 5 to 1 to the first 5 predictors for each of the three measurements of variable importance provided by MaxEnt. Afterwards, we summed the rank to provide an overall metric for variable importance

PC	Rank contribution	Rank permutation	Rank training gain	Overall rank
*bio*4	5	5	4	14
*bio*5	4	2	5	11
*bio*16	3	4	3	10
*NDWIs*	2	3	1	6
*NDWIw*	1	−	2	3

## Additional file

Additional file 1:
**Title of data: R function to perform logistic regression with Bayesian inference.** Description of data: R function to perform logistic regression with Bayesian inference. (PDF 37 kb)
