# Supplementary material for: First assessment of potential distribution and dispersal capacity of the emerging invasive mosquito Aedes koreicus in Northeast Italy
Source: Parasit Vectors. 2016 Feb 3;9:63. doi: 10.1186/s13071-016-1340-9 (PMC4739402; doi:10.1186/s13071-016-1340-9)
Supplement: Additional file 1: — Title of data: R function to perform logistic regression with Bayesian inference. Description of data: R function to perform logistic regression with Bayesian inference. (PDF 37 kb) [file 13071_2016_1340_MOESM1_ESM.pdf]

##
Copyright (C), 2015-2016 Matteo Marcantonio
#
This program is free software; you can redistribute it and/or modify it under
the terms of the GNU General Public License as published by the Free Software
Foundation; either version 2 of the License, or (at your option) any later version.
#
This program is distributed in the hope that it will be useful, but WITHOUT
ANY WARRANTY; without even the implied warranty of MERCHANTABILITY or FITNESS
FOR A PARTICULAR PURPOSE. See the GNU General Public License for more details.
#
A copy of the GNU General Public License is available at
http://www.gnu.org/licenses/gpl.txt, or can be requested to the Free Software
Foundation, Inc., 51 Franklin Street, Fifth
Floor, Boston, MA 02110-1301 USA
#
##

R function logBay for logistic regression with Bayesian Inference

Description:

The function 'logBay' performs logistic regression with Bayesian inference. Currently only simple logistic regressions (no hierarchical structure) with continuous predictor variables are supported.

Usage:

BayesOutput<-logBayes(priorList, respDistr="dbern", inData, orData, numChains, burnIn, numIters, thinVal, backtrans, interaction, posterior, plot)

Arguments:

priorList: a list of character vectors containing priors mean, precision (1/sd) and a parameters defining truncated half distribution (optional).

respDistr: distribution family of the response. Currently only "dbern" is supported

inData: input list with response and predictor variables and a numeric vector with the total number of observations. The latter must be called "N". The response variable column must be named "y"

orData: if the predictor variables in 'inData' are scaled (centred and scaled), 'orData' is specified as the original input list with the same structure of inData and backtrans=TRUE, the model coefficients are transformed in original scale units. Optional.

numChains,burnIn,numIters,thinVal: see ?jags.model for information on these options.

interaction: a character vector of length 2 adding an interaction term. The vector must contain the name of the two interacting variables (e.g., c("x1","x2")). If no interactions are included in the model, it must be set as FALSE. A prior with mean=0 and precision=1e-12 is automatically assigned to the interaction term.

backtrans: if set as TRUE, the model coefficients are back-transformed to the original scale (if the predictor variables are scaled and an orData is provided).

posterior: if TRUE, the posterior distributions are sampled and saved in a list, otherwise only the DIC is calculated and saved.

plot: if TRUE, the posterior distribution of the model coefficients are plotted.

Depends:

coda and rjags package

Value:
a list with the MCMC chains for each model coefficient and the DIC object.

Author:
Matteo Marcantonio

Example:

```
# Synthetic predictors; x1,x2 correlated, x3 independent;
require(MASS)
set.seed(111)
N<-50
M<-matrix(c(-0.7,-0.7,0),3,3) # correlation matrix
diag(M)<-1
X<-mvrnorm(N,c(0,2,0),M,empirical=TRUE)
x1 = X[, 1]
x2 = X[, 2]
x3 = X[, 3]
# y is a linear combinantion of this three variables
z = 1 + x1 + 2*x2 + 5*x3    # linear combination with a bias
pr = 1/(1+exp(-z))  # pass through an inv-logit function
y = rbinom(N,1,pr)  # Bernoulli distr. response variable

# Build the input list
JAGSData <- list(
y = y,
x1 = x1,
x2 = x2,
x3 = x3,
N = N)

# Scale the predictors
JAGSData_scaled <- list(
y = y,
x1 = as.integer(scale(x1)),
x2 = as.integer(scale(x2)),
x3 = as.integer(scale(x3)),
N = N)

# Define the priors
modelTerm <- list(
alpha = c(0,1.0E-12,""),
x1 = c(1.962, 1.5290519881,"I(0, 1.0E8)"),
x2 = c(0, 1.0E-12,""),
x3 = c(0, 1.0E-12,"")
)

# Run the function without interactions
BayesOutput<-logBayes(priorList=modelTerm, respDistr="dbern", inData=JAGSData_scaled,
orData=JAGSData, numChains=4, burnIn=1000, numIters=1000, thinVal=1, backtrans=FALSE,
interaction=FALSE, posterior=TRUE, plot=TRUE)
```

```
    # Run the function with an interaction term
    BayesOutput<-logBayes(priorList=modelTerm, respDistr="dbern", inData=JAGSData_scaled,
orData=JAGSData, numChains=4, burnIn=10000,  numIters=10000, thinVal=1, backtrans=FALSE,
interaction=c("x2","x3"), posterior=TRUE, plot=TRUE)

    # Look at the PPD
    summary(BayesOutput[[1]])
    plot(BayesOutput[[1]],ask=T)

    # Check for convergence
    gelman.diag(BayesOutput[[1]])

## Function code
    logBayes<-function(priorList, respDistr, modelFile = tempfile(), inData, orData, numChains,
burnIn, numIters, thinVal, interaction=FALSE, backtrans=TRUE, posterior=FALSE, plot = FALSE)
{

# Required packages
    require(coda)
    require(rjags)

# Clean data from not needed variables
    termsName<-names(priorList)[which(names(priorList) %in% names(inData))]
    datatest<-(inData[c(termsName,"N","y")])

# Extract values from prior list
    if( length(interaction)>1 ) {
        int = c("0","1e-12","")
        priorList_tmp <- priorList
        priorList_tmp[[length(priorList)+1]] <- int
        names(priorList_tmp)[[length(priorList_tmp)]] <- "int"
        priorVals <- sapply(X = priorList_tmp, FUN = function(inEl) {inEl[1:3]})
    } else {
        priorVals <- sapply(X = priorList, FUN = function(inEl) {inEl[1:3]})
        interaction <- c("","")
        priorList_tmp <- priorList
        }

# Create the model structure
    modelText <- paste(
        "model",
        "{",

# Set a prior distribution for each included covariate
        gsub("^.*?alpha.coef","alpha",paste("\t", names(priorList_tmp), ".coef ~ dnorm(",
priorVals[1,], ", ", priorVals[2,], ")",priorVals[3,], sep = "", collapse = "\n")),
        "\tfor(i in 1:N)",
        "\t{",

# Build the main model
        gsub(".coef [*] alpha[[]i[]]","",paste("\t\tmu[i] <- 1/(1+ exp(-(",
            paste(names(priorList), ".coef * ", names(priorList),"[i]",collapse = " + ", sep = ""),paste("+
```

```
", interaction[1],interaction[2],"[i]",sep=""),")))")),
        paste("\t\ty[i] ~ ",respDistr,"( mu[i] )",sep=""),
        "\t}",
        "}\n",
        sep = "\n")

# Add an interaction term if interaction=TRUE
    ifelse(exists("int"),modelText<-
gsub(paste(interaction[1],interaction[2],"[[]i[]]",sep=""),paste("int.coef
*",interaction[1],"[i]*",interaction[2],"[i]",sep=""),modelText),modelText<-gsub("\\s[\\
+]\\s[[]i[]]","",modelText))

# Print the model specification to the screen
    cat("*** BUGS MODEL SPECIFICATION ***\n")
    cat(modelText)
    cat("*** BUGS MODEL SPECIFICATION ***\n")

# Print the model specification to a temporary file
    cat(modelText, file = modelFile)

# Burn the model in (set the starting values)
    modJAGS <- jags.model(modelFile, data = datatest, n.chains = numChains, n.adapt = burnIn)

# Calculate DIC (Deviance Information Criterion)
    dic<-dic.samples(modJAGS, n.iter = numIters, thin = thinVal, type = "pD")

# Print DIC
    print(dic)

# Run JAGS and draw samples from the posterior
    if( posterior == TRUE)
    {
        ifelse( length(interaction)>1, priorList<-priorList_tmp, priorList<-priorList )

        modOutput <- coda.samples(modJAGS, gsub("alpha.coef","alpha",paste(names(priorList),
".coef", sep = "")), n.iter = numIters, thin = thinVal)

# Gelman plot
        gelman.plot(modOutput)

# Back-transform parameters if interaction=FALSE
        if( interaction[1] == FALSE & backtrans == TRUE ) {
            posterior<-as.data.frame(as.matrix(modOutput))

# Extract chain values
            zb0Sample = posterior[,"alpha"]
            chainLength = length(zb0Sample)
            zbSample = rep(NA,chainLength)
            for (j in names(posterior)[-c(1)]) {
                zbSample = cbind( zbSample, posterior[j])
            }
            zbSample<-zbSample[,-c(1)]
```

```
# Convert back to original scale
            x=orData[termsName]
            y=orData["y"]
            My = mean(as.numeric(y$y))
            SDy = mean(as.numeric(y$y))
            Mx = as.data.frame(as.matrix(lapply(x,mean))[1:length(termsName),1])
            SDx = as.data.frame(as.matrix(lapply(x,sd))[1:length(termsName),1])
            b0Sample = 0 * zb0Sample
            bSample = 0 *zbSample
            for ( stepIdx in 1:chainLength ) {
                b0Sample[stepIdx] = ( zb0Sample[stepIdx]- sum(Mx / SDx *
as.numeric(zbSample[stepIdx,])))
                for ( j in 1:length(termsName)) {
                    bSample[stepIdx,j] = zbSample[stepIdx,j] / SDx[j]
                }
            }

# Save the two rescaled parameters into a data frame
            modOutputRescaled<-as.data.frame(cbind(alpha=b0Sample,bSample))
            names(modOutputRescaled)<-gsub(".coef","",names(modOutputRescaled))
        }
        if (interaction[1] == TRUE & backtrans == TRUE) {
            print(warning("Back-standardization for interaction terms still to be implemented" ))
        }

# Plot results
        if(posterior == TRUE & plot == TRUE) {
              X11()
        plot(modOutput, ask=TRUE)
    }

# Save MCMC samples and DIC in the output file
        if( interaction[1] == FALSE & backtrans == TRUE ) {
            return(list(modOutput,modOutputRescaled,dic,Mx,SDx))
        } else {
            return(list(modOutput,dic))
        }
    }

}
```
